# ESCO2 promotes the proliferation of hepatocellular carcinoma through the PI3K/AKT/ mTOR signaling pathway

**DOI:** 10.7150/jca.112087

**Published:** 2025-06-23

**Authors:** Dapeng Chen, Yue Huang, Weixin Zhang, Youcheng Zhang, Yi Bai, Yamin Zhang

**Affiliations:** 1 First Central Hospital of Tianjin Medical University, Tianjin Medical University, Tianjin 300070, China.; 2 Daping Hospital, Army Medical University, Chongqing 400000, China.; 3 Department of Hepatobiliary Surgery, Tianjin First Central Hospital, Tianjin 300192, China.

**Keywords:** Hepatocellular carcinoma, ESCO2, Cell cycle, Apoptosis, PI3K/AKT/mTOR signaling pathway

## Abstract

**Background:** Establishment of sister chromatid cohesion N-Acetyltransferase 2 (*ESCO2*) is a gene implicated in the establishment of sister chromatid cohesion (SCC) and cell proliferation. We aimed to explore how *ESCO2* affects the proliferation of hepatocellular carcinoma (HCC).

**Methods:** We analyzed ESCO2 expression levels and their association with clinical prognosis using the TCGA, HCCDB, and ICGC databases. Bioinformatics methods were employed to investigate potential regulatory pathways involving ESCO2. CCK-8 assays, colony formation assays, and flow cytometry were used to examine the impact of *ESCO2* knockdown on the malignant biological behavior of HCC cells. Western blotting was utilized to identify the specific regulatory mechanism of ESCO2.

**Results:** *ESCO2* was significantly upregulated in HCC tissues and correlated with a worse prognosis. Bioinformatics analysis revealed that *ESCO2* regulated pathways related to the cell cycle and cell proliferation. Furthermore, knockdown of *ESCO2* significantly inhibited HCC cell proliferation both *in vivo* and *in vitro*. Most importantly, *ESCO2* stimulated the PI3K/AKT/mTOR pathway, which ultimately accelerated the cell cycle and inhibited apoptosis, promoting HCC progression.

**Conclusion:** The present study elucidated the mechanism by which *ESCO2* regulates HCC proliferation: *ESCO2* promotes HCC proliferation by accelerating the cell cycle and inhibiting apoptosis via the PI3K/AKT/mTOR signaling pathway.

## Background

Liver cancer, specifically hepatocellular carcinoma (HCC), is one of the most prevalent solid cancers worldwide, posing a significant global health burden 1, 2. With a relatively low 5-year survival rate of approximately 18%, HCC remains one of the deadliest malignancies 3. The majority of HCC patients are diagnosed at an advanced stage, limiting treatment options to local and systemic therapies, excluding surgery or liver transplantation. Currently, systemic therapy is the standard approach for treating advanced HCC in clinical settings 4, ranging from single-agent targeted therapies (sorafenib or lenvatinib) to combinations of immune checkpoint inhibitors and targeted therapies (atezolizumab plus bevacizumab). Despite significant advancements in systemic therapy, advanced HCC remains a challenging and often fatal disease, given that only a minority of patients achieve durable clinical benefits 5. The carcinogenesis of HCC is now understood to be a complex process, and research has shown that abnormal proliferation is a crucial driver of HCC development 6. Investigating the molecular mechanisms that regulate HCC proliferation will enhance our understanding of its pathogenesis and identify potential therapeutic targets, which may improve patient outcomes in the future.

Establishment of sister chromatid cohesion N-Acetyltransferase 2 (*ESCO2*) is a novel gene implicated in cell proliferation. It belongs to the family of histone acetyltransferases (HATs) that regulate numerous vital biological processes 7. The ESCO2 protein is essential for establishing sister chromatid cohesion (SCC) during the S phase of the cell cycle 8. Sister chromatid cohesion facilitates accurate chromosome segregation, which is crucial for cell cycle progression 9. Previous studies on ESCO2 have revealed that human *ESCO2* mutations result in Roberts syndrome, an inherited developmental disorder characterized by SCC defects and aberrant transcriptional acetylation 10. According to previous research, *ESCO2* is linked to the development of several malignancies, making it a potential biomarker and therapeutic target. *ESCO2* is significantly upregulated in kidney cancer tissues, and *ESCO2* knockdown inhibits cancer cell growth, invasion, and migration by regulating the AKT/mTOR pathway 11. Zhu et al. also demonstrated that *ESCO2* could promote lung adenocarcinoma (LUAD) cell proliferation and metabolic reprogramming of metastasis *in vitro* and *in vivo* 12. However, ESCO2 has been shown to inhibit cancer metastasis in colorectal cancer by reducing MMP2 expression 13. To date, no studies have explored the expression of ESCO2 and its functional role in HCC.

In this study, multi-source high-throughput data analysis revealed that *ESCO2* was significantly upregulated in HCC tissues. Furthermore, HCC patients with high *ESCO2* expression exhibited poorer prognoses. Additionally, *ESCO2* knockdown markedly suppressed HCC cell proliferation both *in vivo* and *in vitro*. Notably, *ESCO2* could promote the PI3K/AKT/mTOR pathway, accelerating the cell cycle and inhibiting apoptosis, thereby increasing HCC growth. Therefore, ESCO2 represents a potential molecular target for HCC.

## 2. Methods and Materials

### 2.1 Data preparation and differential expression analysis

The bulk mRNA sequencing profiles of liver hepatocellular carcinoma (LIHC) and normal liver tissue samples were acquired from the International Cancer Genome Consortium (ICGC, https://dcc.icgc.org/) and The Cancer Genome Atlas (TCGA, https://portal.gdc.cancer.gov/) database. Concurrently, the single-cell RNA sequencing matrix, encompassing 10 tumor tissues and 7 normal tissues, was obtained from GSE149614 (https://www.ncbi.nlm.nih.gov/geo/) 14. Spatial transcriptome sequencing data (HRA000437) 15 was accessed from the Genome Sequence Archive (GSA, https://ngdc.cncb.ac.cn/gsa/). The spatial transcriptomics data of HCC leading-edge sections were selected for further analysis.

TCGA database was initially used to assess the differential expression of ESCO2 mRNA between tumor and normal samples, which contains 374 tumor tissues and 50 adjacent normal tissues. Additionally, the HCCDB database 16, a comprehensive platform(http://lifeome.net/database/hccdb/home.html) incorporating all HCC mRNA sequencing data, was utilized to examine ESCO2 expression levels. In addition, we employed the Seurat R package to process spatial transcriptomics data and performed log-normalization to standardize the data. The expression level and distribution of ESCO2 were visualized on leading-edge sections using Seurat's SpatialFeaturePlot function. Finally, four pairs of HCC and adjacent non-malignant tissue samples were collected from Tianjin First Central Hospital. None of them received preoperative chemotherapy or radiotherapy. All patients provided informed consent for the use of surgical material for academic research and publication. All methods were approved by the Institutional Review Board and the Medical Ethics Committee of Tianjin First Central Hospital.

### 2.2 Survival analysis and functional enrichment analysis

We first excluded samples without follow-up data or with a follow-up time of 0. We then performed survival analysis using the survival R package. Kaplan-Meier survival curves were modeled using the “survfit” function. The optimal cutoff point for gene expression was determined using the "surv_cutpoint" function from the survminer R package. Patients were then divided into two groups based on the optimal cutoff point, and Kaplan-Meier survival curves were modeled for each group. Additionally, we calculated the correlation between clinical data (tumor size, stage, grade, and survival status) and ESCO2 expression levels. The clinical parameters and corresponding ESCO2 expression values of the enrolled patients are listed in Table S2.

To explore the biological functions of ESCO2 in HCC, individuals in the LIHC cohort were divided into ESCO2-high and ESCO2-low subsets based on the median ESCO2 expression value. Gene Set Enrichment Analysis (GSEA) was performed to determine whether predefined gene sets exhibited significant differences between the two subgroups. Two HCC RNA-seq datasets, TCGA-LIHC and ICGC, were utilized to identify ESCO2-related regulatory genes. In each dataset, individuals were divided into ESCO2-high and ESCO2-low subsets based on the median ESCO2 expression value. Subsequently, a differential expression analysis was conducted to identify differentially expressed genes (DEGs) between the ESCO2-high and ESCO2-low subgroups (*p* < 0.05, log FC ≥ 1). The association between ESCO2 and these DEGs was then determined using Spearman's correlation analysis in both datasets (*p* < 0.05, correlation coefficient ≥ 0.3). Intersecting genes from these closely related gene sets were identified as ESCO2-related regulatory genes. Finally, the functional roles of these regulatory genes were elucidated using the Metascape online platform.

### 2.3 Single-cell analysis

All cells were filtered through quality control, retaining cells with RNA counts ranging from 200 to 7000 and mitochondrial gene expression percentages less than 5%. Data were normalized using the "LogNormalize" approach with a scale factor of 10,000. Seurat's CellCycle Scoring function was employed to determine each cell's cell cycle score. The influence of UMI counts and mitochondrial content was regressed using Seurat's ScaleData function. Subsequently, batch effects were eliminated using the Harmony R package. The top 30 principal components and the top 2000 variable genes were selected for cell clustering and uniform manifold approximation and projection (UMAP) visualization. Canonical marker genes identified in previous studies were utilized to annotate cell types. DEGs were identified using the FindMarkers function. Gene Ontology (GO) and Kyoto Encyclopedia of Genes and Genomes (KEGG) pathway enrichment analysis for DEGs was performed using clusterProfiler R packages 17. Additionally, Gene Set Variation Analysis (GSVA) was employed to assess the expression scores of 50 hallmark pathways among cell clusters.

### 2.4 Cell culture and transfection

Huh7 and MHCC97H cell lines were obtained from ICell Bioscience Inc. (Shanghai, China). All hepatoma cell lines were cultured in DMEM medium (Gibco BRL Life Technologies Inc., USA) supplemented with 10% fetal bovine serum (FBS) (Gibco BRL Life Technologies Inc., USA) and 1% penicillin-streptomycin (HyClone, CA, USA) at 37°C. To ensure effective suppression of ESCO2 expression, two independent small interfering RNAs (siRNAs) were designed. The siRNAs were ordered from GenePharma, and their sequences are listed in Table S1. Hepatoma cell lines were transfected with siRNA using a transfection reagent (Thermo Fisher Scientific, United States) according to the manufacturer's protocol when cells reached 70-80% confluence in the culture plate.

The shRNA constructs targeting ESCO2 and negative controls were obtained from GenePharma. Huh7 and MHCC97H cell lines were infected with lentiviral particles expressing the ESCO2-targeting shRNA, following the manufacturer's protocol. Stable transfectants were selected using 10 μg/mL puromycin (Sigma-Aldrich, St. Louis, MO, USA). The sequence of the shRNA targeting ESCO2 was 5'-GCAAATCAAGGCTCACCAT-3'.

### 2.5 RT-qPCR and western blotting

The cells were harvested for RNA and protein extraction after 48 and 72 hours of siRNA transfection, respectively. Total RNA was extracted using TRIzol reagent (Invitrogen). RNA quantification was performed using the One-Step SYBR PrimeScript RT-PCR kit (Takara, Japan), and cDNA was synthesized using the cDNA Reverse Transcription Kit. *ESCO2* expression was normalized to *GAPDH* and calculated using the 2^-ΔΔCt^ method. The primers used are listed in Table S1 (Sangon Biotech).

Cells were harvested and incubated with RIPA lysis buffer (Thermo Fisher Scientific, USA) supplemented with a protease inhibitor cocktail (Roche, Switzerland) on ice for 30 minutes to extract cellular proteins. Tumor tissue proteins were isolated using the Tissue Protein Extraction Reagent (Thermo Fisher Scientific, USA) according to the manufacturer's protocol. Protein concentration was measured using the BCA assay (Thermo Fisher Scientific, USA). Equivalent amounts of total protein from each group were separated by sodium dodecyl sulfate-polyacrylamide gel electrophoresis (SDS-PAGE) and transferred to a nitrocellulose membrane. After blocking with 5% non-fat milk for 1 hour, primary and secondary antibodies (Table S1) were applied according to standard Western blotting procedures. Protein bands were visualized using SuperKine™ West Femto Maximum Sensitivity Substrate (Abbkine) and detected with an imaging system (Tanon, Shanghai, China). The grayscale intensity of protein bands was quantified using ImageJ software. All antibodies used are listed in Table S1.

### 2.6 Cell proliferation and colony formation assay

For the CCK-8 experiment, 2000 cells were seeded into a 96-well plate, transfected, and cultured for a specified duration. Each well received 10 µL of CCK-8 reagent. After a 2-hour incubation, the absorbance at 450 nm was measured. To evaluate colony formation capacity, 1000 cells were seeded into 6-well plates and cultured for 12 days. Cells were fixed and stained when visible colonies appeared. The number of colonies in each well was then counted.

### 2.7 Ethynyl-2-Deoxyuridine (EdU) incorporation assay

Cell proliferation was assessed using an EdU imaging analysis kit (Abbkine, Wuhan, China). 50,000 cells were seeded per 15 mm glass-bottom culture dish and transfected. Following transfection, the culture medium was removed, and a suitable volume of EdU (ApexBio Technology LLC) was added according to the manufacturer's instructions. Cells were incubated for 2 hours, then fixed and stained with DAPI (Sigma-Aldrich, USA) for nuclear counterstaining. The percentage of EdU-positive cells (red) was determined using a fluorescence microscope (Nikon, Tokyo, Japan).

### 2.8 Flow cytometry related to cell cycle and apoptosis

Both normal and transfected cells were collected and washed. Fluorescent labeling dyes, Annexin V and PI (Beyotime), were employed for apoptosis labeling and incubated for 30 minutes. Individual cells were analyzed for distinct fluorescence intensities using a flow cytometer (BD Biosciences). Data were processed with FlowJo software to obtain the percentage of apoptotic cells.

Cells were trypsinized, resuspended in a complete growth medium, and washed with PBS. Following overnight fixation in 70% cold ethanol, cells were stained using the Cell Cycle and Apoptosis Analysis Kit (Beyotime). Cell cycle distribution was determined using a flow cytometer (BD Biosciences), and data were analyzed and processed using FlowJo.

### 2.9 *In vivo* proliferation experiment

All animal procedures were approved by the Animal Ethics Committee of Tianjin Medical University. Four-week-old female BALB/c-nude mice were purchased from SPF (Beijing) Biotechnology Co., Ltd. 1 × 10⁷ MHCC97H cells with stable *ESCO2* knockdown or control RNA were subcutaneously injected into the right flank of each nude mouse (n = 5 per group). On day 21, mice were euthanized, and tumors were excised. Tumor tissues were fixed, paraffin-embedded, sectioned, and stained with anti-Ki67 antibody (dilution 1:200, Affinity BioSciences, Cincinnati, OH, USA). Slide images were examined and quantified by a pathologist.

### 2.10 Statistical analysis

Analyses between the two groups were performed using the Wilcoxon test, while the one-way analysis of variance (ANOVA) test was utilized for comparisons involving three or more groups. All statistical calculations were performed using R Studio and GraphPad software.

## 3. Results

### 3.1 ESCO2 is overexpressed in HCC tissues and associated with poor prognosis

**Figure 1:**
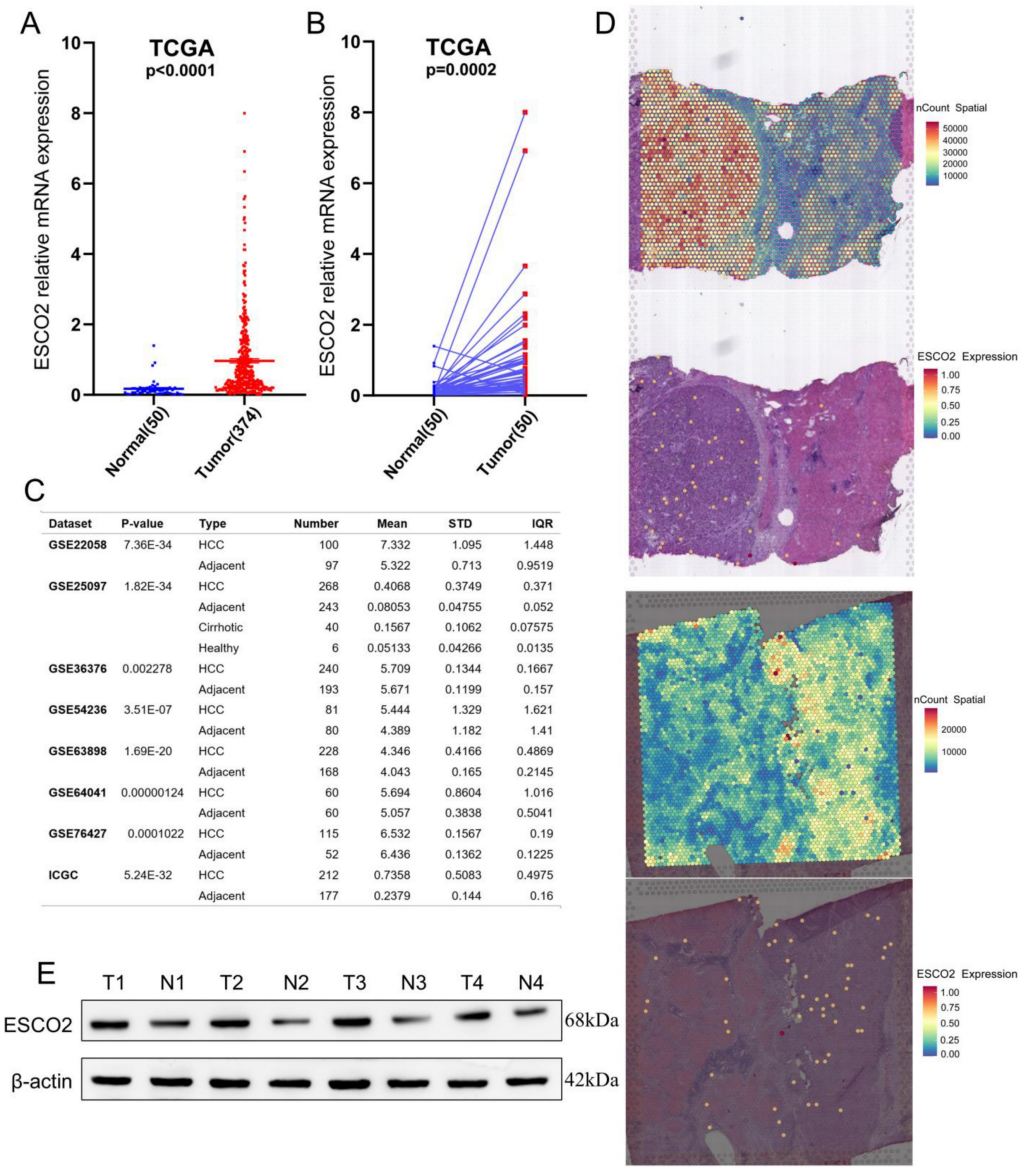
** ESCO2 is overexpressed in liver cancer tissues.** (A) Comparison of ESCO2 expression level between HCC and normal samples in TCGA database. (B) Comparison of ESCO2 expression levels between 50 paired HCC tissues and corresponding normal tissues. (C) Multiple HCC sequencing data from the HHCDB database was utilized to determine the expression levels of ESCO2 in HCC and normal tissues. (D) Spatial plots show the spatial expression pattern of ESCO2 in this study. (E) Western blot protein detection of the ESCO2 expression levels in adjacent normal tissues and paired HCC tissues. The optical density ratio of bands represent objective proteins to band of β-actin was calculated.

Previous studies have demonstrated that *ESCO2* is markedly upregulated in lung, kidney, and colorectal malignancies 11-13; however, no studies have focused on ESCO2 expression in HCC. Using TCGA data, it was first identified that HCC samples exhibited significantly higher *ESCO2* mRNA levels than normal tissues (Figure 1A). *ESCO2* was significantly overexpressed in most HCC tissues using RNA-seq data from 50 matched cohorts of HCC and paracancerous tissues (Figure 1B). HCCDB database analysis of eight independent HCC study cohorts indicated that ESCO2 expression was significantly higher in HCC tissues than normal ones (Figure 1C). Meanwhile, spatial transcriptomic data from two HCC leading-edge sections was employed to further confirm the overexpression of *ESCO2* in HCC tissues (Figure 1D). Finally, the protein level of ESCO2 in HCC samples was significantly higher than in paracancerous samples (Figure 1E). These findings demonstrated that HCC tissues exhibited significant overexpression of ESCO2 compared to nearby non-tumor tissues.

**Figure 2:**
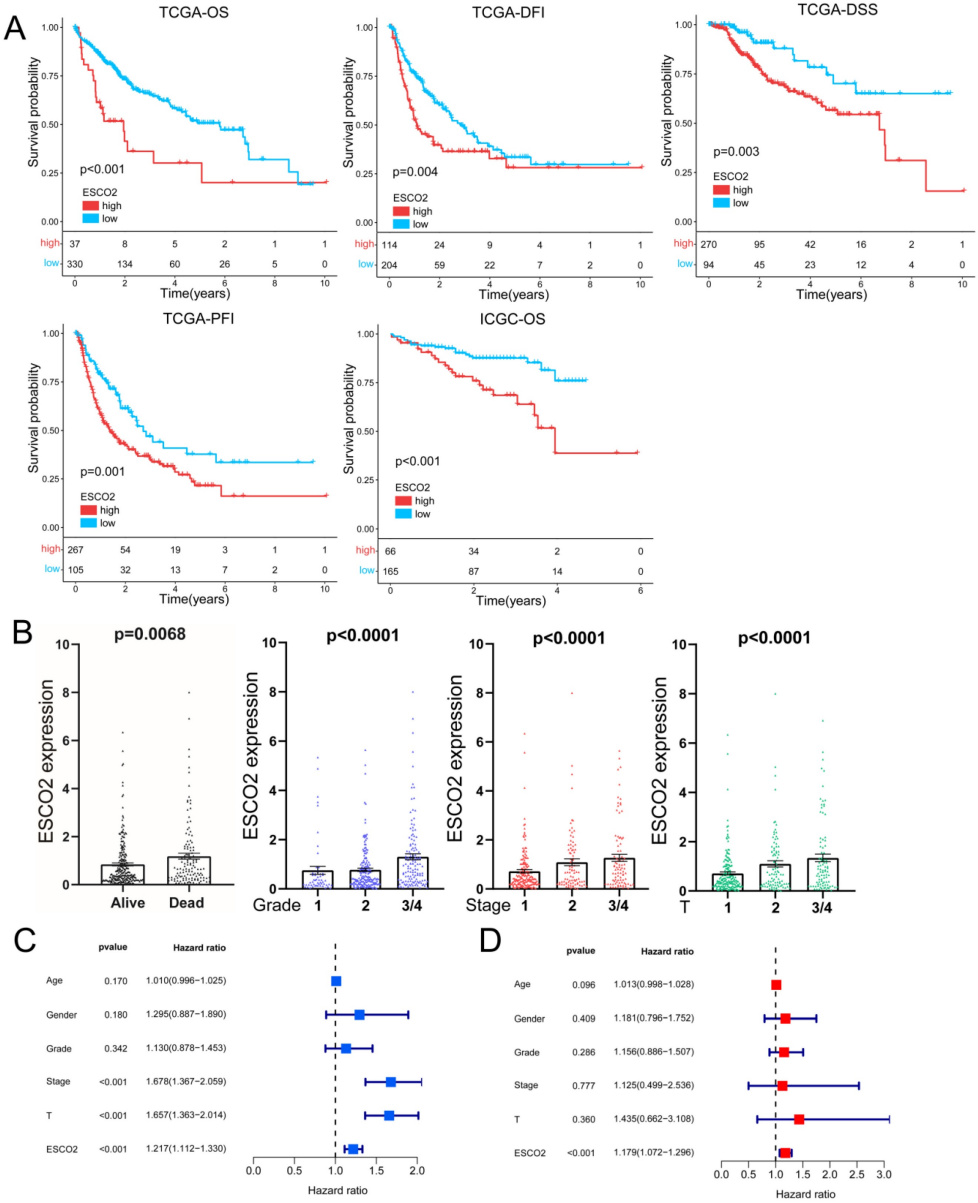
** ESCO2's Prognostic Value in HCC.** (A) The Kaplan-Meier survival curves stratified by ESCO2 expression level. The indicators include OS, DFI, DSS, and PFI. (B) Association between ESCO2 expression and tumor stage, tumor metastasis, tumor grade and survival state. (C) Univariate and multivariate(D) analyses showed that ESCO2 expression was a prognostic indicator independent of other clinical characteristics.

The potential of ESCO2 as a prognostic biomarker was then explored, given its overexpression in HCC tissues. Kaplan-Meier survival analyses for HCC patients were performed using TCGA data to determine the relationship between *ESCO2* expression and prognosis, focusing on overall survival (OS), disease-free interval (DFI), disease-specific survival (DSS), and progression-free interval (PFI). Upregulated *ESCO2* expression was significantly associated with poor outcomes (Figure 2A). Next, the association between survival outcomes and *ESCO2* expression was evaluated in the ICGC cohort. Similarly, HCC patients with increased *ESCO2* expression demonstrated poor OS (Figure 2A). To further substantiate the involvement of ESCO2 in cancer progression, the association between *ESCO2* expression and clinical data was evaluated. *ESCO2* expression value positively correlated with tumor stage, size, grade, and survival status (Figure 2B). The most common clinical indicators for assessing the progression of cancer are tumor stage, size, and grade. Our analysis confirmed the positive correlation between ESCO2 and theses clinical indicators, implying that ESCO2 contributes to HCC progression. Furthermore, univariate and multivariate Cox regression analyses revealed that ESCO2 expression was an independent prognostic indicator independent of other clinical factors (Figure 2C and 2D). Overall, our findings suggest ESCO2 as a potential promoter of HCC initiation and progression.

### 3.2 ESCO2 regulates the cell cycle

**Figure 3:**
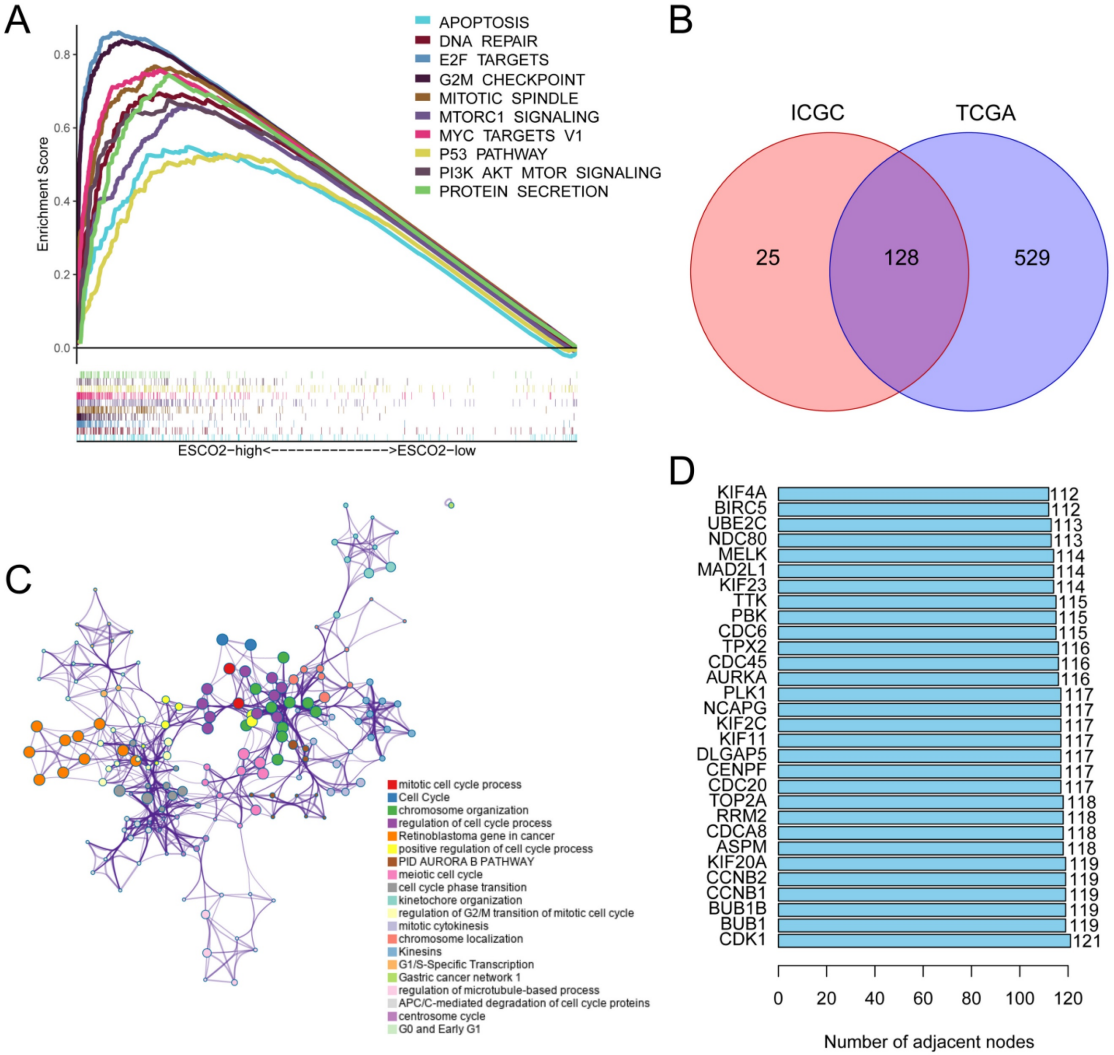
** ESCO2 regulates the cell cycle.** (A) The GSEA analysis between ESCO2-high and ESCO2-low groups in HCC. Each panel's left and right sides represent the enriched pathways of the ESCO2 high and low expression group, respectively. (B) The intersecting genes of DEGs between the ESCO2-high and ESCO2-low groups. (C) The ESCO2-related regulatory gene functions were mainly enriched in mitotic cell cycle and cell cycle. The interactive network was constructed using the Metascape online platform. (D) Bar plot of the top 30 genes with the largest number of adjacent nodes in the PPI network of ESCO2-related regulatory genes.

Our study demonstrated that ESCO2 is a reliable biomarker for HCC and further investigated the effector function of ESCO2 in tumorigenesis. To explore the oncogenic role of this protein, HCC patients were divided into ESCO2-high and ESCO2-low subsets based on the median ESCO2 expression value. Gene Set Enrichment Analysis (GSEA) was performed to identify gene sets enriched in both groups. The top 10 most significant enriched pathways were visualized in this study. For the ESCO2-high subset, upregulated genes demonstrated enrichment in the G2M checkpoint, PI3K-AKT-MTOR signaling, and mTORC1 signaling pathways, which are associated with cell proliferation and cell cycle progression. Additionally, the apoptosis and p53 pathway was also significantly enriched in the ESCO2-high subset (Figure 3A). To further investigate the regulatory pathways of ESCO2, TCGA and ICGC data were utilized to identify ESCO2-related regulatory genes, as described in the methods section. A total of 128 ESCO2-related regulatory genes were identified (Figure 3B). These genes showed enrichment in the cell cycle, mitotic cell cycle process, cell cycle phase transition, and mitotic cytokinesis (Figure 3C). To identify core genes, a protein-protein interaction (PPI) network of ESCO2-related regulatory genes was constructed using the STRING database 18. The top 5 core genes were *CDK1*, *BUB1*, *BUB1B*, *CCNB1*, and *CCNB2*, all of which encode essential proteins for regulating the cell cycle (Figure 3D). Cyclin-dependent kinase 1 (CDK1) is essential for facilitating the G2/M transition, controlling the G1 process, and governing the G1-S transition 19.

Numerous studies have demonstrated that CDK1 dysregulation leads to chromosomal instability, aggressive tumor growth, and accelerated cell proliferation 20. CDK1 inhibitors are potential effective anti-cancer small-molecule drugs. For instance, in an HCC patient-derived xenograft (PDX) tumor model, treatment with the CDK1 inhibitor RO3306 in combination with sorafenib significantly decreased tumor growth 21. Similarly, BUB1 and BUB1B are crucial for maintaining accurate chromosome segregation and reducing aneuploidy formation during mitosis 22. Experiments have shown that BUB1 and BUB1B can promote cancer cell proliferation and are promising therapeutic targets 23-25. The appropriate regulation of the G2/M transition phase depends on the presence of CCNB1 (Cyclin B1), located on chromosome 5q13.2. CCNB1 is aberrantly expressed in several malignancies and is highly associated with patient prognosis 26, 27. HCC tissues exhibit significantly higher levels of CCNB1, and HCC cell proliferation can be suppressed by *CCNB1* knockdown 28. In conclusion, our bioinformatics analysis suggests that ESCO2 regulates pathways associated with the cell cycle.

### 3.3 Single-cell analysis of ESCO2

**Figure 4:**
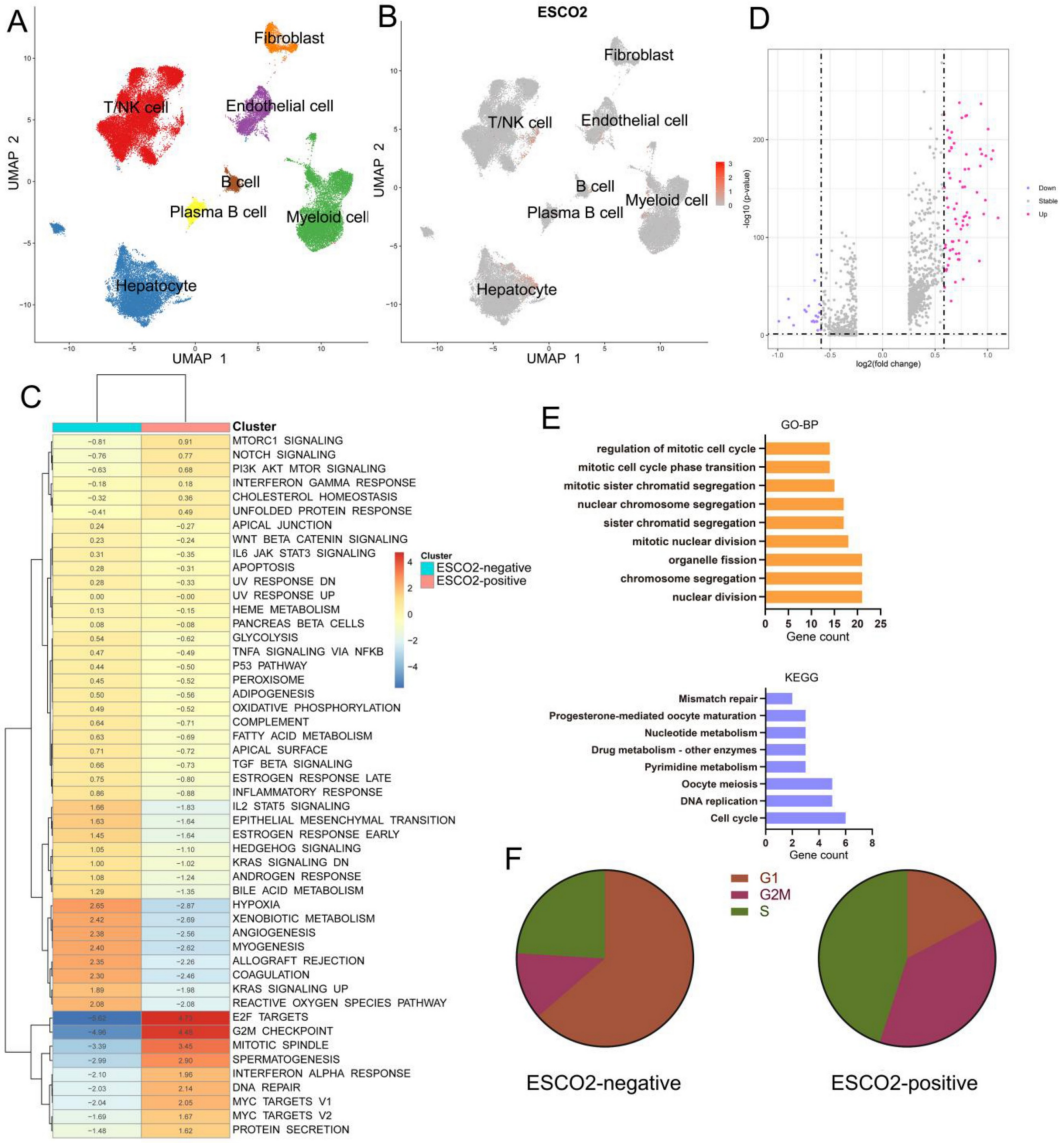
** single cell analysis of ESCO2 in HCC.** (A) UMAP plots of all single cells of HCC patients, showing all cell types in the plot. (B) UMAP plot showing the expression of ESCO2 in each cell types. (C) Differences in hallmark pathway activities scored with GSVA. The t values calculated by a linear model are shown. (D) Volcano plot of the DEGs between ESCO2-positive hepatoma cells and ESCO2-negative hepatoma cells. The upregulated genes (log2(fold change) >0.5) are colored red, while the downregulated genes (log2(fold change) less than 0.5) are colored blue. (E) GO (up) and KEGG (bottom) analysisof the DEGs between ESCO2-positive hepatoma cells and ESCO2-negative hepatoma cells. FDR <0.05 was considered significantly enriched. (F) Pie charts of the proportions of cells in each stage of the cell cycle in ESCO2-positive and ESCO2-negative hepatoma cells.

The tumor microenvironment (TME) is critical for tumor development and immune escape of tumor cells 29. Stromal and immune cells within the TME are genetically stable, unlike tumor cells. Modifying TME components holds promise for overcoming the pervasive issue of immunotherapy resistance. As a result, current cancer research has shifted from a cancer cell-centric to a non-tumor cell-centric approach 30. Due to the high heterogeneity and plasticity of the TME, oncogenes may play distinct roles in each cell type. Traditional bulk RNA sequencing data may not be sufficient to accurately assess the functional roles of oncogenes. To address this limitation, we employed single-cell analysis to explore the effector function of ESCO2 in malignant cells at the single-cell resolution. We found that myeloid cells, B cells, T/NK cells, and malignant cells expressed ESCO2 (Figure 4A and 4B). Hepatoma cells were then extracted and divided into ESCO2-positive and ESCO2-negative subgroups based on ESCO2 expression levels. GSVA results showed that E2F targets, G2M checkpoint, and MYC targets signaling pathways were enriched in ESCO2-positive cells, which aligns with our previous bioinformatics results (Figure 4C). Differential expression analysis was performed between ESCO2-positive and ESCO2-negative subgroups (Figure 4D). GO analysis revealed that the DEGs were primarily enriched in terms associated with proliferation, including regulation of the mitotic cell cycle, nuclear division, and sister chromatid segregation. Furthermore, DEGs showed enrichment in DNA replication and cell cycle pathways in KEGG analysis (Figure 4E). Subsequently, when the cell cycle profiles of the two groups were compared, a higher proportion of ESCO2-positive group cells were observed in the G2/M phase, suggesting a more robust capacity for proliferation (Figure 4F).

### 3.4 ESCO2 knockdown inhibits the growth of HCC cells* in vitro* and* in vivo*

**Figure 5:**
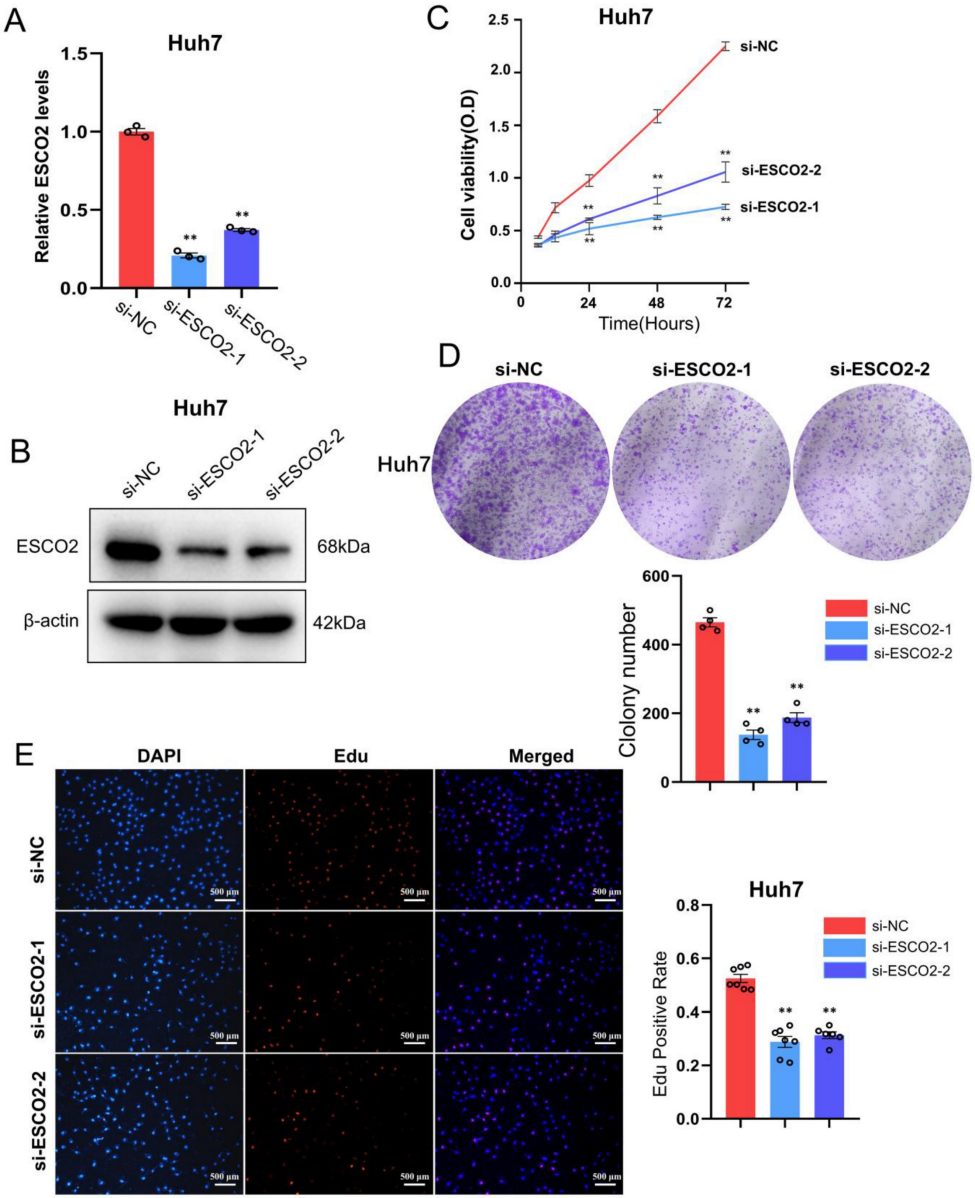
** ESCO2 promotes Huh7 cell proliferation *in vitro.*** ESCO2 knockdown efficiency was confirmed by qPCR and western blotting (B) in Huh7 cell lines. (C) CCK8 analysis of cell viability in ESCO2-knockdown HCC cells at 0, 24, 48, and 72h, respectively, compared with the si-NC group. (D) Colony formation assay showed that ESCO2-knockdown inhibited cell proliferation in Huh7 cell lines. (E) EdU proliferation assay in Huh7 cells transduced with si-NC, si-ESCO2-1 or si-ESCO2-2. Scale bar = 500 μm.

**Figure 6:**
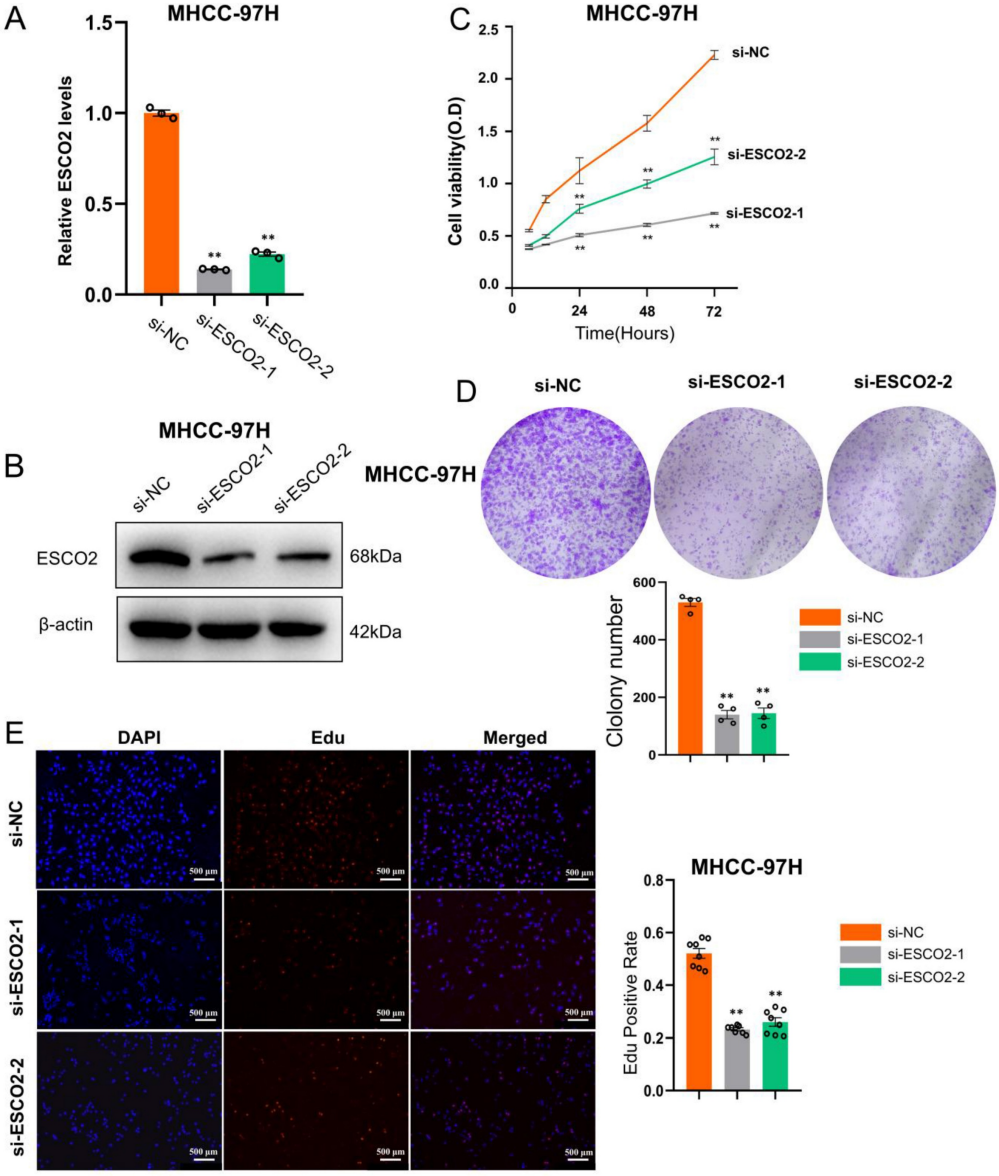
** ESCO2 promotes MHCC97H cell proliferation *in vitro.*** ESCO2 knockdown efficiency was confirmed by qPCR and western blotting(B) in MHCC97H cell lines. (C) CCK8 analysis of cell viability in ESCO2-knockdown MHCC97H cells at 0, 24, 48, and 72h, respectively, compared with the si-NC group. (D) Colony formation assay showed that ESCO2-knockdown inhibited cell proliferation in MHCC97H cell lines. (E) EdU proliferation assay in MHCC97H cells transduced with si-NC, si-ESCO2-1 or si-ESCO2-2. Scale bar = 500 μm

We first validated the knockdown efficiency of ESCO2-siRNA in hepatoma cell lines. Western blotting and PCR results confirmed that two siRNAs were effective in downregulating ESCO2 at both the mRNA and protein levels (Figure 5A and 5B). After confirming *ESCO2*'s knockdown efficiency, several *in vitro* experiments were conducted to examine the impact of *ESCO2* knockdown on the biological behavior of HCC cells. First, the CCK-8 assay revealed that in Huh7 cell lines, the absorbance of the siRNA1# and siRNA2# groups was significantly lower than the si-NC group at 24 h, 48 h, and 72 h (Figure 5C). Similarly, the colony formation assay demonstrated that at day 11, a significantly smaller number of cell colonies were formed following *ESCO2* knockdown (Figure 5D). Intriguingly, the EdU assay revealed that the proliferation of HCC cells was significantly inhibited by *ESCO2* knockdown (Figure 5E). Similarly, the knockdown of *ESCO2* in MHCC97H cells significantly inhibited its proliferative ability (Figure 6A-E). The results presented above collectively indicated that ESCO2 knockdown suppressed the proliferative capacity of hepatoma cell lines.

**Figure 7:**
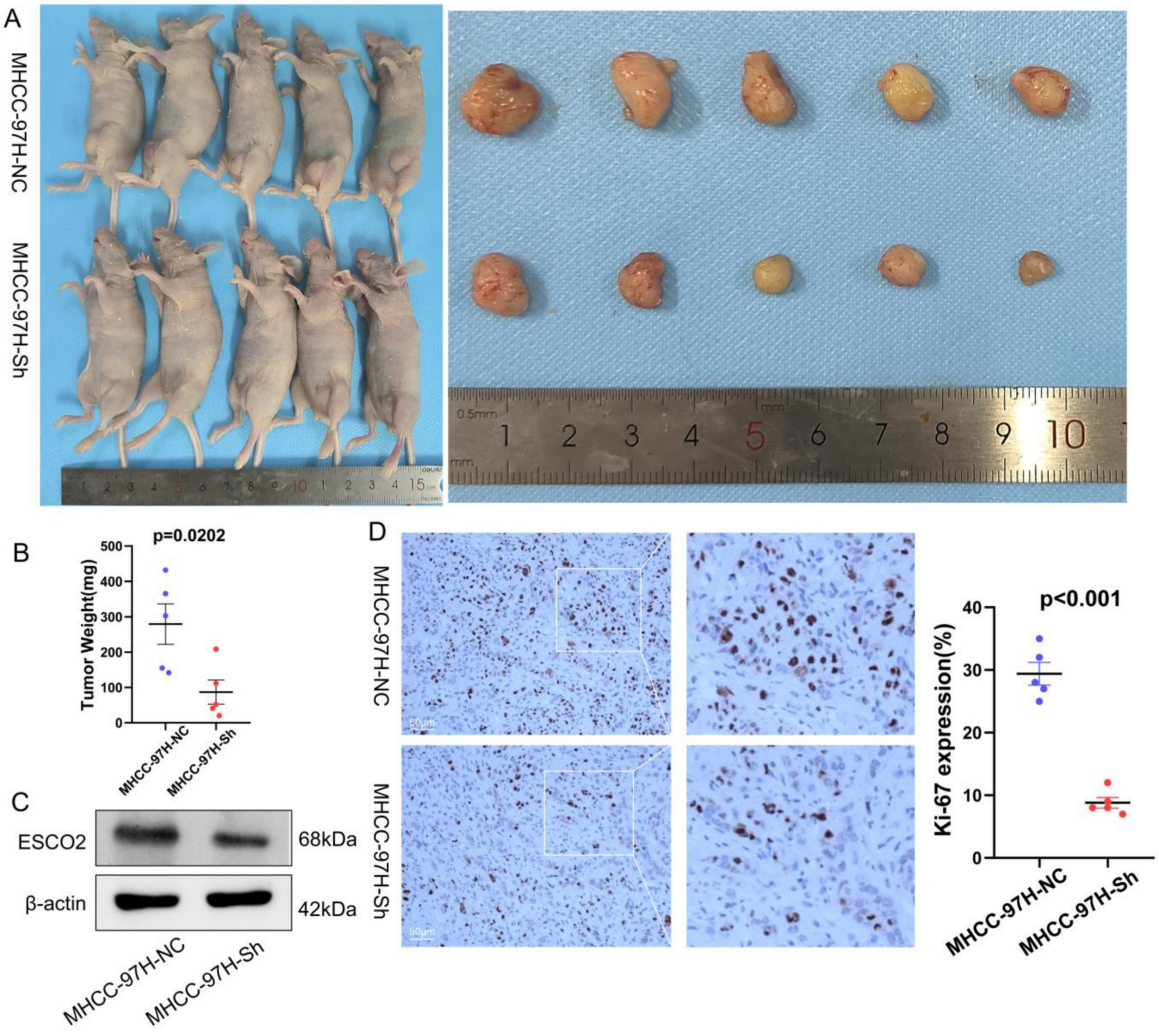
** ESCO2 promotes MHCC97H cell proliferation *in vivo.*** (A) The images of the subcutaneous tumors formed in nude mice between the scramble and ESCO2-knockdown groups. (B) Comparing the tumor weight of the ESCO2-knockdown and scrambling groups statistically. (C) ESCO2 was efficiently knocked down in tumor tissue formed in nude mice, as determined by western blotting. (D) IHC analysis of the xenograft tumor tissues' Ki-67 expression levels in the ESCO2-knockdown and scramble groups.

Due to the higher knockdown efficiency of siRNA-1, we then constructed a lentivirus using the sequence of siRNA-1 for subsequent experiments. We then constructed a subcutaneous xenograft mouse model to examine the effects of regulating ESCO2 expression on HCC cell proliferation *in vivo.* MHCC97H cells stably transfected with sh-ESCO2 or sh-Control were injected into the flanks of nude mice, and tumor volumes were recorded periodically. Tumor volume and weight were significantly reduced in the sh-ESCO2 group compared to the sh-Control group (Figure 7A and 7B). Tumors were then excised for immunohistochemical (IHC) analysis. The results showed that the Ki-67 level was substantially lower in mice injected with ESCO2-knockdown MHCC97H cells compared to the corresponding control mice (Figure 7C and 7D). Taken together, these findings demonstrate that ESCO2 could promote the growth of HCC cells both *in vivo* and *in vitro*.

### 3.5 Knockdown of ESCO2 inhibits HCC cell proliferation by inducing G1-S cell cycle arrest and stimulating apoptosis *in vitro*

**Figure 8:**
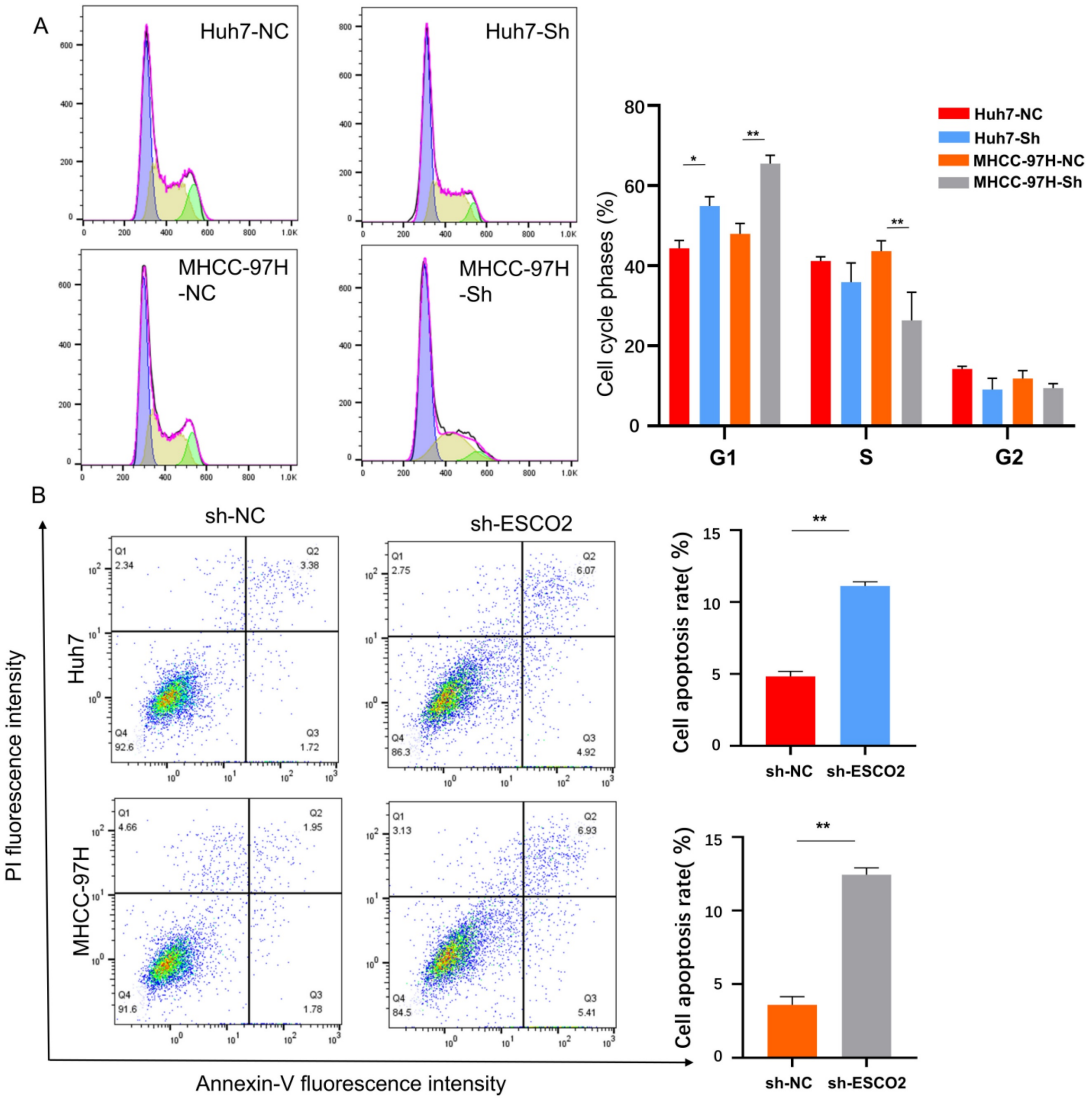
(A) Results and statistics of cell cycle-related flow cytometry for HCC cells in the sh-NC group and sh-ESCO2 group. (B) Flow cytometry results and statistics related to apoptosis of HCC cells.

According to our bioinformatics investigation, ESCO2 primarily participates in cell cycle regulation. Therefore, flow cytometry was performed to analyze the cell cycle distribution of HCC cells after *ESCO2* knockdown. Compared to the sh-NC group, the sh-ESCO2 group exhibited a significantly larger percentage of cells in the G1 phase (Figure 8A). This finding indicated that *ESCO2* knockdown could induce S-phase arrest in HCC cells. Additionally, apoptosis-related flow cytometry results revealed that the proportion of apoptotic cells in both the MHCC97H and Huh7 cell lines was significantly higher in the sh-ESCO2 group compared to the sh-NC group (Figure 8B). Consequently, *ESCO2* knockdown could induce apoptosis in HCC cells.

### 3.6 Investigation of the mechanism by which ESCO2 regulates the development of HCC cells

**Figure 9:**
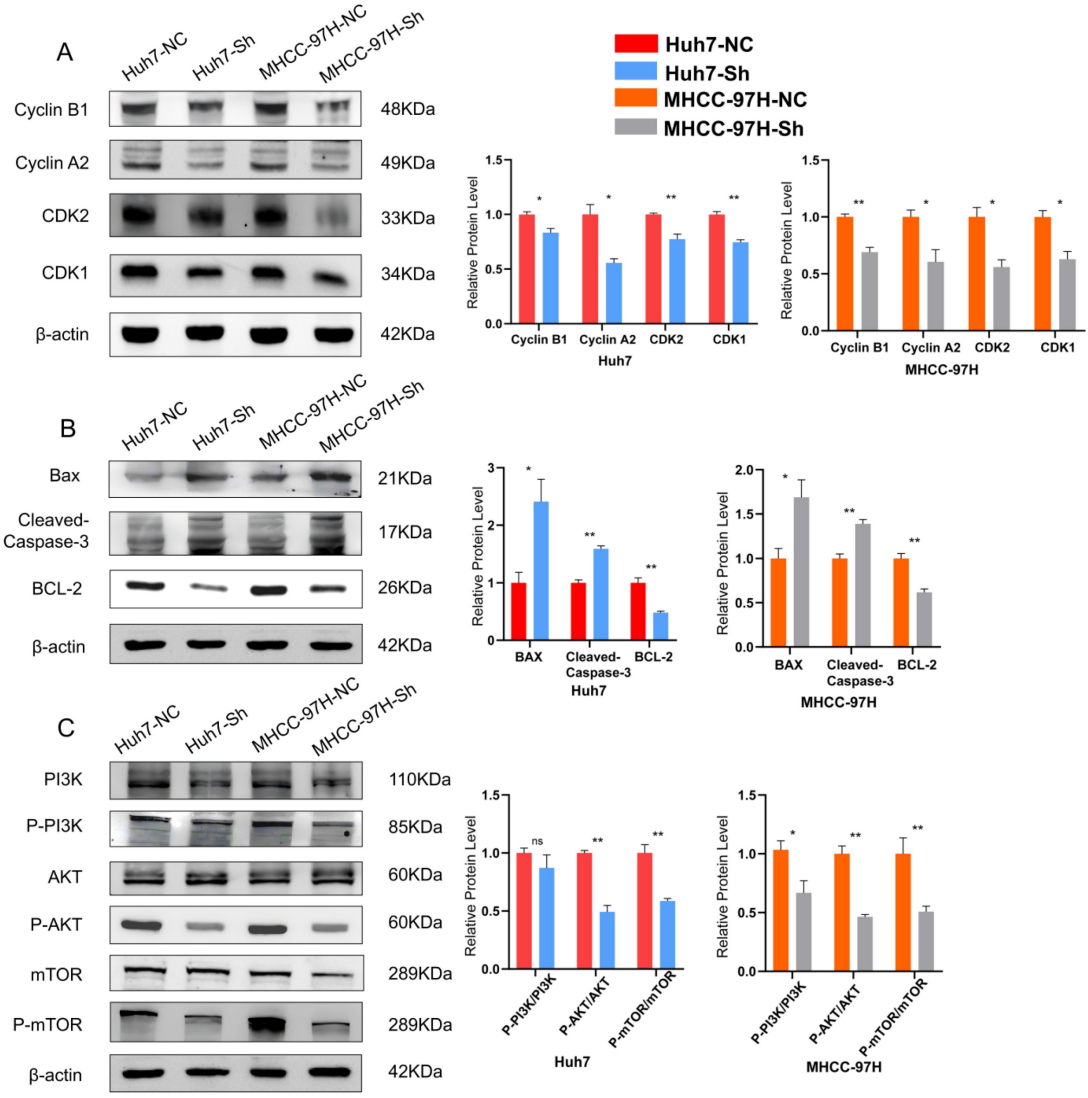
** ESCO2 regulates HCC proliferation via the PI3K/AKT/mTOR signaling pathway.** (A) Western blot results and statistics of cell cycle-related proteins of HCC cells. (B) Western blot results and statistics of cell apoptosis-related proteins of HCC cells. (C) Western blot analysis showing the levels of P-PI3K, P-AKT and P-mTOR when ESCO2 was knocked down.

We subsequently investigated the specific oncogenic mechanism of ESCO2 after confirming that *ESCO2* knockdown prevents HCC cell growth by inducing G1-S phase cell cycle arrest and apoptosis. First, in Huh7 and MHCC97H cell lines, Western blotting of cell cycle-related proteins revealed that the sh-NC group exhibited increased levels of CDK1 and cyclin B1 expression compared to the sh-ESCO2 group. Additionally, CDK2 and cyclin A2 protein expression was significantly decreased after ESCO2 knockdown (Figure 9A). These findings suggest that *ESCO2* knockdown induces cell cycle arrest by regulating the CDK1/CDK2 signaling pathways. Subsequently, Western blotting of apoptosis-related proteins demonstrated that inhibition of *ESCO2* significantly increased the expression levels of pro-apoptotic proteins (BAX and Caspase-3). Conversely, compared to the sh-NC group, the sh-ESCO2 group displayed significantly decreased expression of the anti-apoptotic protein BCL-2 (Figure 9B). Hence, *ESCO2* knockdown can trigger apoptosis in HCC cells by regulating these apoptosis-specific proteins. Meanwhile, considering the GSEA results suggesting that *ESCO2* might be involved in the regulation of the PI3K/AKT/mTOR signaling pathway, and given the frequent activation of the PI3K/AKT/mTOR pathway in cancer and its crucial role in regulating cell cycle and apoptosis, we investigated how *ESCO2* knockdown affected the PI3K/AKT/mTOR pathway. The band intensities of phosphorylated PI3K, AKT, and mTOR were significantly weaker in sh-ESCO2 cells compared to control cells, indicating that knockdown of *ESCO2* significantly inhibited the PI3K/AKT/mTOR pathway (Figure 9C). In summary, ESCO2 may influence the expression of proteins involved in the cell cycle and apoptosis by activating the PI3K/AKT/mTOR signaling pathway, which supports the growth of HCC cells.

## Discussion

Advanced HCC is characterized by a poor prognosis. Despite significant advancements in systemic therapy that have increased survival time in this population, the median overall survival remains extremely short. As the current first-line treatment for advanced HCC, atezolizumab/bevacizumab therapy has demonstrated superior efficacy and tolerability compared to sorafenib 31. Other drug combination treatment options are currently being tested. Therefore, the effective combination of immunotherapy and targeted medicine is a research highlight to further improve the prognosis of advanced HCC patients. Advancements in sequencing technology present greater possibilities for identifying diagnostic and therapeutic targets that could benefit HCC patients through early detection, precise treatment, and prognostic monitoring. Cell division and cell death are fundamental processes that govern organism growth and development. Both processes are abnormally regulated in cancer, leading to uncontrolled proliferation 32. Uncontrolled cancer growth and metastasis are the primary causes of liver cancer's high mortality and recurrence rates. Understanding the mechanisms underlying HCC proliferation would aid in discovering innovative and more effective therapeutic strategies to improve survival rates. In previous studies, ESCO2 was identified as a potential target for cancer therapy, as it is a pivotal protein in the cell division process. Acetylation of the SMC3 subunit of the cohesin protein by ESCO2 acetyltransferase facilitates SCC. The cohesion of the cohesin protein complex between sister chromatids ensures accurate chromosomal segregation 33, 34. *ESCO2* was significantly elevated in tumor tissues in several malignancies, and *ESCO2* knockdown could inhibit cancer cell proliferation, invasion, and migration. However, the underlying mechanism of *ESCO2* upregulation in cancer development remains incompletely understood. The potential of *ESCO2* as a therapeutic target for HCC is unknown. Our research elucidates the mechanism by which *ESCO2* promotes HCC proliferation. Accordingly, *ESCO2* may serve as a valuable molecular diagnostic marker and potential therapeutic target for HCC patients.

Our study provided hitherto undocumented evidence that *ESCO2* expression was significantly upregulated in HCC tissues using extensive HCC sequencing data. Leveraging public databases, our data included 1446 HCC tissues and 943 normal tissues, enhancing the robustness of our findings. More importantly, we identified that HCC samples possessed a significantly higher ESCO2 protein level than paracancerous samples. Given the high expression of *ESCO2* in HCC tissues, it may function as an oncogene that promotes hepatocarcinogenesis. Furthermore, upregulated ESCO2 expression was strongly associated with worse outcomes in HCC patients. ESCO2 expression positively correlated with tumor grade, size, and stage. To some extent, tumor stage and grade represent the progression of liver cancer. Hence, ESCO2 may significantly contribute to HCC progression. Finally, we verified that ESCO2 was an independent prognostic indicator for HCC patients. Based on the aforementioned findings, ESCO2 is a reliable biomarker for HCC patients. Oncogenes are known to impact the biological behavior of cancer cells (migration, invasion, and proliferation), thereby facilitating cancer progression. Bioinformatic analysis suggested that ESCO2 expression was closely related to hepatocarcinogenesis and development. Therefore, we employed siRNA to inhibit *ESCO2* expression in HCC cell lines. Our cellular experiments supported the conclusion that *ESCO2* knockdown significantly suppressed the proliferative ability of HCC cell lines. Additionally, the subcutaneous xenograft nude mouse model confirmed that ESCO2 could promote the growth of HCC cells *in vivo*. Consequently, we confirmed that *ESCO2*, a potential oncogene, may promote HCC growth and lead to a poor prognosis by inducing the malignant biological behavior of HCC cells. Previous studies have shown that ESCO2 could enhance the proliferative and migratory capacities of lung, kidney, and stomach cancer cells. Our findings aligned with these previous reports.

Oncogenes typically regulate cancer cell biology by modifying cellular signaling pathways. Previous research found that ESCO2 affects the AKT/mTOR pathway in kidney cancer 11. *ESCO2* knockdown inhibited mTOR/RPS6K1 activation and upregulated AMPKα and p53 phosphorylation in gastric cancer cells 35. In lung cancer, ESCO2 acetylates hnRNPA1, maintaining it in the nucleus and ultimately enhancing aerobic glycolysis by increasing PKM2 and decreasing PKM1 expression 12. In the present study, we first explored the effector function of ESCO2 in HCC using bioinformatic methods. GSEA results revealed that ESCO2 participates in E2F targets, G2M checkpoint, PI3K-AKT-MTOR signaling, and mTORC1 signaling pathways, which are associated with cell cycle and proliferation. We also identified 128 ESCO2-related regulatory genes. These genes showed enrichment in the cell cycle, mitotic cell cycle process, cell cycle phase transition, and mitotic cytokinesis. Moreover, our study explored the biological function of ESCO2 at the single-cell level, which avoids the interference of other non-tumor cells. Consistent with the results of bulk RNA sequencing data analysis, cell proliferation-associated pathways, such as E2F targets, G2M checkpoint, and MYC targets signaling, were significantly enriched in ESCO2-positive hepatoma cells. Overall, our bioinformatic analysis suggests that ESCO2 plays a significant role in regulating cell cycle and cell proliferation-related pathways.

We next explored ESCO2's role in regulating the cell cycle and cell growth through a series of experiments. Flow cytometry demonstrated a significantly larger percentage of HCC cells in the G1 phase after *ESCO2* knockdown. Besides, *ESCO2* knockdown regulated the expression levels of HCC cell cycle proteins (CDK1, cyclin B1, CDK2, and cyclin A2), resulting in cell cycle arrest at the G1 phase. Cyclin B1 is a crucial cyclin that regulates the G2/M phase. During the S and G2 phases of the cell cycle, cyclin B is synthesized in significant amounts, enters the nucleus, binds to CDK1, and activates the G2 to M phase transition 26. Similarly, cyclin A2 (CCNA2) is a regulator of essential CDK protein kinases. Cyclin A2 binds and activates CDK2, thereby promoting the transition through the G1/S and G2/M phases 36. Hence, *ESCO2* knockdown induces cell cycle arrest by regulating the CDK1/CDK2 signaling pathway. Human malignancies frequently exhibit cell cycle dysregulation, and targeting cell cycle proteins is one of the most promising areas for cancer therapy. Scientists have demonstrated the effectiveness of targeting proteins associated with the cell cycle to limit tumor growth. For instance, researchers identified that homoharringtonine (HHT) could bind to the PPI site of CDK2, disrupting the interaction between CDK2 and cyclin A, resulting in a loss of CDK2 activity and protein degradation, and preventing tumor progression 37. Additionally, all three CDK4/6 inhibitors have been approved by the United States Food and Drug Administration (FDA) for breast cancer treatment 38. Therefore, ESCO2 may be a promising target for cell cycle-related protein targeting. Furthermore, we discovered that *ESCO2* knockdown induced apoptosis in HCC cells. Western blotting results showed that knockdown of *ESCO2* increased the levels of apoptosis-related proteins (BAX, Caspase-3), promoting apoptosis in HCC cells. Apoptosis is a crucial step in the death of tumor cells. Evasion of apoptosis contributes to cancer cell escape and treatment resistance. Consequently, many cancer therapies rely on successful apoptotic induction 39. Numerous drugs targeting apoptosis-related proteins have been developed with promising outcomes 40. Furthermore, our research revealed that ESCO2 regulates cell cycle and apoptosis by activating the PI3K/AKT/mTOR pathway. The PI3K/Akt/mTOR signaling pathway is indispensable in many cellular biological processes, including cell proliferation, survival, metabolism, motility, angiogenesis, and response to stress and therapy. Extensive research supports its critical role in regulating tumor growth, metabolism, metastasis, and treatment resistance 41, 42. Small-molecule inhibitors targeting key kinases in the PI3K/Akt/mTOR pathway have been developed and evaluated in preclinical models and human clinical trials. For instance, the FDA approved idelalisib, a PI3K inhibitor, to treat follicular B-cell non-Hodgkin's lymphoma and relapsed chronic lymphocytic leukemia 43. Currently, there are over 35 clinical trials targeting the PI3K/Akt/mTOR pathway in HCC. However, outcomes indicated that the clinical benefit of monotherapy with these inhibitors remains limited 44. This may be due to the interaction of the PI3K/Akt/mTOR pathway with multiple signaling pathways, increasing the likelihood of adverse events and treatment resistance. Combination therapy may be a more effective strategy for advanced HCC. Understanding the crosstalk and feedback with other pathways will, therefore, be key challenges in targeting the PI3K/Akt/mTOR pathway. Future studies should explore the upstream and downstream regulatory mechanisms of the PI3K/Akt/mTOR pathway, which will provide crucial insights for developing novel therapeutic targets or proposing innovative combination therapeutic regimens. Our study will provide valuable insights for the development of drugs targeting the PI3K/Akt/mTOR pathway.

Herein, we determined that *ESCO2* was significantly upregulated in HCC tissues and linked to a poorer prognosis. Knockdown of *ESCO2* significantly inhibited HCC cell proliferation both *in vivo* and *in vitro*. Most notably, ESCO2 could promote the PI3K/AKT/mTOR pathway, accelerating the cell cycle and inhibiting apoptosis, and thus increasing HCC growth. These findings offer innovative and valuable insights for targeted therapy in HCC.

However, this study has some limitations that should be acknowledged. First, investigations into the specific mechanism of ESCO2's involvement in HCC remain insufficient, which is a focus for future studies. Additionally, our study lacks a real-world clinical cohort to assess the predictive power of ESCO2 for prognosis in HCC.

## Conclusion

In summary, our study reveals for the first time the molecular mechanism by which *ESCO2* promotes HCC proliferation. *ESCO2* may activate the PI3K/AKT/mTOR pathway, thereby accelerating the cell cycle and inhibiting apoptosis, and consequently increasing HCC growth. Overall, this work identifies a novel HCC target gene, providing valuable insights for HCC prognostic assessment and molecular therapy.

## Supplementary Material

Supplementary table 1 and table 2 legend.

Supplementary table 2.
