# Supplementary material for: ESCO2 promotes the proliferation of hepatocellular carcinoma through the PI3K/AKT/ mTOR signaling pathway
Source: J Cancer. 2025 Jun 23;16(9):2929–45. doi: 10.7150/jca.112087 (PMC12244334; doi:10.7150/jca.112087)
Supplement: Supplementary file 1 — Supplementary table 1 and table 2 legend. [file jcav16p2929s1.pdf]

**Table S1.** The antibodies, primers, oligonucleotides and shRNAs used in this study are shown.

| **Table S1. The antibodies, primers, oligonucleotides and shRNAs used in this study are shown.** | | | |
|---|---|---|---|
| **Antibodies** | | | |
| anti-ESCO2 Ab | 23525-1-AP | Rabbit | Proteintech |
| anti-P-mTOR Ab | 2971S | Rabbit | Cell Signaling Technology(CST) |
| anti-mTOR Ab | 66888-1-Ig | Mouse | Proteintech |
| anti-P-AKT Ab | S473 | Rabbit | CST |
| anti-AKT Ab | 60203-2-Ig | Mouse | Proteintech |
| anti-P-PI3K Ab | 17366 | Rabbit | CST |
| anti-PI3K Ab | 20584-1-AP | Rabbit | Proteintech |
| anti-β-actin Ab | 60008-1-Ig | Mouse | Proteintech |
| anti-CDK1 Ab | 19532-1-AP | Rabbit | Proteintech |
| anti-CDK2 Ab | 60312-1-Ig | Mouse | Proteintech |
| anti-Cyclin A2 Ab | 66391-1-Ig | Mouse | Proteintech |
| anti-Cyclin B1 Ab | 55004-1-AP | Rabbit | Proteintech |
| anti-BCL2 Ab | 26593-1-AP | Rabbit | Proteintech |
| anti-BAX Ab | 60267-1-Ig | Mouse | Proteintech |
| anti-Caspase3 Ab | 66470-2-Ig | Mouse | Proteintech |
| goat anti-mouse HRP-conjugated IgG | | SA00001-1 | Proteintech |
| goat anti-rabbit HRP-conjugated IgG | | SA00001-2 | Proteintech |
| **Primers name** | | | |
| ESCO2 (Q-PCR) | Forward | CACTGGGACGCACCCAAAA | |
| | Reverse | CACTTGCCTTGTCGCAAAAG | |
| GAPDH (Q-PCR) | Forward | CAGGAGGCATTGCTGATGAT | |
| | Reverse | GAAGGCTGGGGCTCATTT | |
| **Oligonucleotides** | | | |
| si-NC | sense:5'-UUCUCCGAACGUGUCACGUTT-3' | | |
| si-ESCO2-1 | sense:5'-GCAAAUCAAGGCUCACCAUTT-3' | | |
| si-ESCO2-2 | sense:5'-CUCUUAGACCAGGAUUAUCTT-3' | | |
| Control shRNA | sense:5'-TTCTCCGAACGTGTCACGT-3' | | |
| sh-ESCO2 | sense:5'-GCAAATCAAGGCTCACCAT-3' | | |

**Table S2.** The clinical parameters and corresponding ESCO2 expression value of enrolled patients in this study.
